# Supplementary material for: The ontology of the anatomy and development of the solitary ascidian Ciona: the swimming larva and its metamorphosis
Source: Sci Rep. 2020 Oct 21;10:17916. doi: 10.1038/s41598-020-73544-9 (PMC7578030; doi:10.1038/s41598-020-73544-9)

**The ontology of the anatomy and development of the solitary ascidian *Ciona*: the swimming larva and its metamorphosis**

Authors: Kohji Hotta^1^*, Delphine Dauga^2^ & Lucia Manni^3^*

^1^ Department of Biosciences and Informatics, Faculty of Science and Technology, Keio University, Kouhoku-ku, Yokohama 223-8522, Japan, khotta@bio.keio.ac.jp

^2^ Bioself Communication, 28 rue de la bibliotheque, 13001 Marseille, France, contact@bioself-communication.com

^3^ Department of Biology, University of Padova, Padova, Italy, lucia.manni@unipd.it

**Supplementary Figure S1**

Title of data: Life cycle of *Ciona*

Description of data: Scheme of the life cycle of *C. robusta* (*C. intestinalis* type A). Blue box: developmental stages described in Hotta et al., 2007. Red box: developmental stages described in this paper.

**
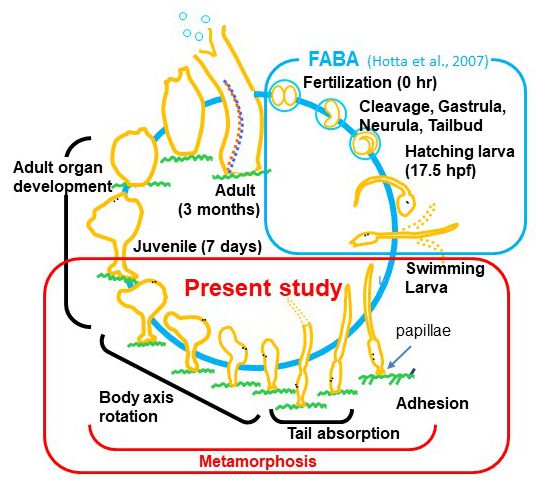
**

**Supplementary Data S2**

Title of data: Description of features in each developmental stage. Each entity, both developmental and anatomical, is written in bold when introduced for the first time; relations between entities are in italics; entity definitions between quotation marks; ID in brackets. Abbreviations used in the cited Figures and Supplementary Figures are in italics in brackets.

**Stage 26**

This is the **Hatching Larva Stage** (CirobuD:0000049; 17.5 h, Supplementary Figure S9). The larva has a roundish trunk and immature papillae (*pp* in S9A, B, D^I^; CirobuA:0000675) with round tips; it exhibits irregular **tail** (CirobuA:0000797) movements. Eight strips of **epidermal cells** (*epi* in S9B, B’, C; CirobuA:0000594) constitute the **tail epidermis** (CirobuA:0000738). The larval **pharynx** (*pha* in S9B; CirobuA:0000682) shows a narrow lumen ^1^. The larval nervous system is subdivided into a central and peripheral nervous system. The former, as described above, contains several entities, for example, the otolith and the ocellus with **lens cells** (CirobuA:0000648), **photoreceptors** (CirobuA:0000683), and **pigment cup cells** (CirobuA:0000685); and the visceral ganglion (*vg* in S9B) with its motor neurons. The **larval peripheral nervous system** (CirobuA:0000681) is differentiating and each epidermal sensory neuron, *i.e.*, the **Rostral Tail Epidermal Neurons** (*RTEN* in S9A; CirobuA:0000709), the **Dorsal Caudal Epidermal Neurons** (*DCEN* in S9A; CirobuA:0000589), and the **Ventral Caudal Epidermal Neurons** (*VCEN* in S9A; CirobuA:0000757), shows dendritic arbors. A vacuolated notochord (*noto* in S9B, B^I^, D) extends as an elongated structure in the tail. The **oral siphon primordium** (*osp* in S9B; CirobuA:0000670) is in the form of an epidermal invagination not yet communicating with the pharynx lumen. The **atrial siphon primordia** (*rasp* and *lasp* in S9C^I^; CirobuA:0000368), the pair of invaginations of the dorsal-lateral trunk epidermis, called also **atrial placodes** (CirobuA:0000908), are not yet open to the exterior. The trunk is covered by a double-layered **tunic** (CirobuA:0000750) that, at this stage, is not well distinguishable at the histological level: the inner compartment of the tunic (CirobuA:0000882), bordered by the **inner cuticular layer** (CirobuA:0000883), and the **outer compartment of the tunic** (CirobuA:0000884), bordered by the **outer cuticular layer** (CirobuA:0000885).

**Stage 27**

This corresponds to the **Early Swimming Larva** **Stage** (CirobuD:0000050; 17.5-20 h; Fig. 2). The larva makes regular tail movements while swimming. The trunk elongates along the anterior-posterior axis. The **postpharyngeal tract** (*post pha* in Fig. 2C^II^; CirobuA:0000871) is developing from the posterior pharynx and the **endodermal strand** ^2^ (*est* in Fig. 2C^III^) is histologically recognizable.

The **endostyle primordium** (*est* in C^III^; CirobuA:0000617), *developing from* A7.1, A7.2, A7.5, B7.1, and B7.2 cells ^1^, is recognizable in the anterior pharynx (*ant pha* in Fig. 2B^II^). A wider lumen of the pharynx can be observed (compare *pha lum* in B of Supplementary Figure S9 and Fig. 2C^II^ E^I^). The atrial siphon primordia indent in the **posterior lateral trunk** **epidermis** (a7.14 and a7.15 cell lines) (CirobuA:0000916; compare *last* and *rasp* in C^I^ in Supplementary Figure S9 with Fig. 2D^II^, E, E^II^). Mesenchyme cells (*mech* in Fig. 2B^I^, C^I^, C^II^, D) in the posterior ventral trunk become round. Among sensory structures in the sensory vesicle (*sv* in B^I^, C, C^I^, C^II^), the **coronet cells** (*cor* in Fig. 2B^II^; CirobuA:0000890) can also be noted. These cells, forming a hydropressure organ ^3,4^, are considered by some authors to be the homolog of the vertebrate hypothalamus ^5–8^.

**Stage 28**

This is the **Mid-Swimming Larva** **Stage** (CirobuD:0000051; 20-22 h; Supplementary Figure S10). The papillae (*pp* in S10A-C^II^) elongate and their basal part expands. The trunk is square in shape. The ciliary network belonging to the epidermal sensory neurons (**ascidian dendritic network in tunic**, or ASNET; CirobuA:0000892) becomes more complex (*ATEN*, *DCEN*; *RTEN*, and *VCEN* in S10A). The **preoral lobe** (CirobuA:0000901; *pl* in S10B), a wide anterior body cavity between the pharynx and the anterior epidermis, is recognizable. Here, round mesenchyme cells are present (*mech* in S10B). Spaces among the epithelia (**haemocele**, CirobuA:0000888) become larger; here, mesenchyme cells represent the **haemocyte** (CirobuA:0000571; synonym of blood cell) precursors. According to Parrinello and co-authors ^8^, haemocytes in adult animals are stem cells and granulocytes. The latter include clear granulocytes (precursors to clear vesicular granulocytes), microgranulocytes, and vacuolar granulocytes (including unilocular granulocytes and globular granulocytes). **Tunic cells** (CirobuA:0000751) are also recognizable in the inner compartment of the tunic. They differentiate from mesenchyme cells. Coronet cells are well-differentiated on the left side of the sensory vesicle. The **otolith** (CirobuA:0000671) senses gravity and gravitaxis is observed at this stage ^9^.

**Stage 29**

This is the **Late Swimming Larva** **Stage** (CirobuD:0000052; 22-24 h; Supplementary Figure S11). With respect to the previous stage, the trunk is longer and narrower, and the tail is longer. Moreover, the trunk profile is squared at the trunk-tail transition (S11A, C-C^I^); in cross-section, the trunk and its tunic (*tun* in S11D) are polygonal and star-shaped, respectively (see S11 D-D^vii^). All of the larval structures for swimming are fully mature: the **tunic fin** (*tf* in S11A, D^IIV^; CirobuA:0000622) located along the dorso-ventral axis, the **tail muscle** (*tmc* in S11D^IIV^; CirobuA:0000739) fibers, and the tunic ciliated sensory fields (*ATEN*, *DCEN*; *RTEN*, and *VCEN* in S11A-B;Terakubo et al., 2010; Yokoyama et al., 2014). In *Ciona*, the larva swims by tail locomotion for several hours. The gravitaxis and visual behaviors are tightly interconnected at this stage ^9^. The duration of the swimming period is variable among individuals. In our observations, it lasts until 23.6 hpf on average, *i.e.*, until the adhesion at the beginning of metamorphosis.

The **gut primordium** (synonym: “gut rudiment”; *gp* in S11D^VI^; CirobuA:0000862) is histologically recognizable as endodermal tissue posterior to the pharynx. Here, the **protostigmata** (CirobuA:0000870) rudiments are now recognizable (*psm* in S11C; ^2^). According to Hirano and Nishida (2000), in *Halocynthia roretzi*, the atrial epithelium *develops from* A7.2, A7.1, A7.5, and B7.1 cell lines. It gives rise to the paired **atrial siphons** (CirobuA:0000366), not yet in communication with the **atrial cavities** (CirobuA:0000798) (*lasp* and *rasp* in S11C, D^VI^).

**Stage 30**

The **Adhesion Stage** (CirobuD:0000053) regards the larva attaching to a suitable substrate through its adhesive papillae (24-27 h; Fig. 3). The papillae change significantly after adhesion (Fig. 3A-C), becoming deflated and sometimes curved. They also start to degenerate. At this stage, the adhesion area is flat. Also called **holdfast** ^1^, in the successive stages it will elongate in the **stalk** (CirobuA:0000632), the epidermal peduncle by which the juvenile is attached to the substratum. The stalk possesses a cavity, derived from the preoral lobe (*pl* in Fig. 3B, B^I^, C^I^), with its own **blood** (CirobuA:0000571) circulation.

The two atrial siphons are now in continuity with the atrial cavities. In the juvenile (Stage 41), the siphons will fuse, becoming a single, dorsal atrial siphon. The sensory organs are recognizable within the sensory vesicle but are beginning to degenerate (*ot* and *oc* in C^III^ and C^vi^).

The inner and outer compartment of the tunic, with their inner and outer cuticular layer, respectively, are well recognizable (*ict* and *oct*, *iclt1*(*C1)* and *oclt*(*C2*), respectively, in Fig. 3C-C^I^).

**Stage 31**

At the **Early Tail Absorption Stage** (CirobuD:0000054) 27-8 hpf; Supplementary Figure S12 A-B^I^), the shrinkage of the **tail epidermis** (*epi* in S12B^I^; CirobuA:0000738) begins at the tail tip (**Tail tip epidermis**: CirobuA:0000949) (compare S12B^I^with S9B^I^). In the same time, the tail epidermal cells, originally flat, change into thick and cuboidal (*epi* in S12B^I^). The actin staining in the posterior tail is relatively strong, indicating the actin’s involvement in the shrinkage process ^10^. In addition, the tail inner tissues, such as the notochord (*noto* in S12B), the endodermal strand (*est* in S12D^I^), and the muscles (*tmc* in S12D^I^), begin to be arranged irregularly in the posterior tail region. In some individuals, the tail slightly bends at the trunk-tail transition (Fig. 1B, Stage 31). The otolith and ocellus are still present, although the larval brain is degenerating; their remnants will be recognizable during the Juvenile Period. The papillae, with their **dorsal palp neurons** (a8.18 line; CirobuA:0000601) and **ventral palp neurons** (a8.20 line; CirobuA:0000771), are no longer histologically recognizable at the end of the stage (*End stage*).

**Stage 32**

This corresponds to the **Mid Tail Absorption Stage** (CirobuD:0000055) 28-29 h; Supplementary Figure S12C-F^III^), when 50% of the tail has been resorbed into the larval trunk. The tail is shorter and thicker than in the previous stage (*deg tail* in S12C). The tail muscles (*tmc* in S12D^I^, F^I^) contract together with the notochord; they fold and stack into the posterior trunk (, *abs tail* in S12F^I-II^). On the other hand, the **tail epidermis** (CirobuA:0000738) contracts without folding and finally invaginates into the trunk region. The process recalls the one described during the absorbing of the tail of *Halocynthia roretzi* and *Botryllus schlosseri* ^11,12^. Although the contribution of apoptosis is not excluded ^13^, it has been suggested that the tail epidermis and the extracellular **notochord sheath** (CirobuA:0000946) generate the strong forces retracting the axial organs into the trunk ^12^. In fact, the tail absorption is inhibited by cytochalasin B, indicating that actin fibers play an important role in the process ^14^.

Concomitantly with tail absorption, the ASNET lose their organization ^15^.

The gut continues its differentiation. The **oral siphon** (*osp* in S12D^I^, F^I^; CirobuA:0000668) opens. The **oesophagus** (*oes* in S12F^II^; CirobuA:0000621) ^2,16^, the **stomach** (CirobuA:0000737) ^1,2,17^, and the **intestine** (CirobuA:0000635) ^2^ are histologically well recognizable. Also, the **heart** (CirobuA:0000628) is visible ^18–22^.

The **test cells** (CirobuA:0000915) are no longer present. They were originally encased in superficial depressions of the developing oocyte by the vitelline coat ^17,22–24^. After fertilization, they were moved into the perivitelline space, to attach to the outer cuticular layer of the tunic. At Stage 32, they are eliminated together with the outer tunic compartment layer and the outer cuticular layer. The stalk starts to elongate.

**Stage 33**

This corresponds to the **Late Tail Absorption Stage** (CirobuD:0000056; 29-30 h; Supplementary Figure S13), during which the tail becomes completely absorbed. Together with the tail, the 88 larval entities associated with the embryonic and larval stages are no longer recognizable (Fig. 6).

Both the notochord and the tail muscles are folded several times and coiled into the posterior trunk (*abs tail* in S13A-A^I^, B-B^I^). Moreover, the posterior trunk epidermis wraps around the absorbing tail. Strong actin staining can still be observed in the absorbing axial organ (muscles and notochord) and in the degenerating tail epidermis.

In histological sections, several newly formed structures associated with the juvenile lifestyle can be defined. Some of them are the **body wal**l (CirobuA:0000857), the **atrial siphon muscles** (CirobuA:0000367), and the **pericardial cavity** (CirobuA:0000678) ^16,20,21,25^. The stalk continues to elongate. Tunic cells *(tunc* in S13B) are numerous in the definitive tunic (the original inner compartment of the tunic with its inner cuticular layer).

**Stage 34**

At the **Early Body Axis Rotation Stage** (CirobuD:0000057; 30-36 hpf; Fig. 4), the stalk (Fig. 4A, B^III^) continues to elongate and forms, with the endostyle axis, an angle of about 90° (Fig. 1B, Stage 34). The strong actin intensity in the trunk region associated to the tail remnants (*tail remn* in Fig. 4A^I^, A^IV^) indicates that the latter is tightly packed.

Adult organs proceed with their differentiation. In the digestive system, the **pyloric caecum** (*pyc* in Fig. 4B^II^; CirobuA:0000630) is differentiating evagination of the stomach (*stom* in Fig. 4A^II^, B^III^-B^IV^). The latter starts to enlarge.

The **oral cavity** (CirobuA:0000802), representing the **oral siphon lumen** (*os* in A^III^, B-B^I^), extends to the rim of the **velum** (*ve* in Fig. 4B; CirobuA:0000900) and **tentacles** (CirobuA:0000741, now recognizable ^26–29^. The oral siphon lumen is in continuity with the **branchial chamber** (*brc* in Fig. 4A^II-IV^, B^I-II^; CirobuA:0000863; synonym: branchial sac, branchial cavity) lumen. Tunic cells are also in the tunic covering the inner oral siphon epidermis.

**Stage 35**

At the **Mid Body Axis Rotation Stage** (CirobuD:0000058; 6-45 h; Supplementary Figure S14), the endostyle axis is perpendicular to the axis passing through the stalk (Fig.1B, Stage 35). In the branchial chamber (*brc* in S14A^I-IV^), which is more expanded than in the previous stage, one pair of elliptical **gill-slits** (*lpsm* and *rpsm* in S14A^II^; CirobuA:0000623), separated by a **transverse bar** (CirobuA:0000746), allows for filtration. The transverse bar contains the transverse sinus of the branchial sac. The **peripharyngeal band** (CirobuA:0000680), which is the ciliated band of the pharynx delimiting the **prebranchial zone** (CirobuA:0000869) from the branchial one, is visible ^30^.

**Stage 36**

At the **Late Body Axis Rotation Stage** (CirobuD:0000059) 45-60 h (2 dpf); Fig. 5), the angle between the endostyle axis and the axis passing through the stalk is 30°- 60° (Fig. 1B, Stage 36). With respect to the previous stage, some new entities are now recognizable. At the gut level, the **pyloric gland** (*pg* in Fig. 5D^I^; CirobuA:0000705) emerged from the pyloric caecum. At the neural system level, the **neural complex** (CirobuA:0000659), composed of the **cerebral ganglion** (CirobuA:0000582), and the **neural gland complex** (CirobuA:0000661) are distinguishable. The latter is formed of the **neural gland body** (*ng* in Fig. 5B, D), which anteriorly exhibits a gland aperture, the **ciliated funnel** (CirobuA:0000584). The latter is located in the **dorsal tubercle** (CirobuA:0000932), on the roof of the prebranchial zone. Posteriorly, the neural gland body elongates into the **dorsal strand** (*dst* in Fig. 5D; CirobuA:0000930). In the adult, a **dorsal strand plexus** (CirobuA:0000931) extends along the dorsal strand. Some **nerves** (CirobuA:0000929) are elongating from the neurons located in the cerebral ganglion.

The filter-feeding activity starts at this stage. Consequently, multiple entities linked to the respiratory and alimentary tract become physiologically functioning. Food, brought by water entering the oral siphon, reaches the branchial cavity (*brc* in Fig. 5A^I^, B-C, D^I^) (delimited by the **branchial epithelium** (CirobuA:0000677)) and passes through the oesophagus (*oes* in Fig. 5D^I^), the stomach (*stom* in Fig. 5A^I-II^, B-B^I^, D^II^) and the intestine (divided into the **proximal** (*prox int* in Fig. 5D^II^; CirobuA:0000872), **mid** (*mint* in Fig. 5D^I^; CirobuA:0000655), and **distal intestine** (CirobuA:0000631)) for digestion. Fecal pellets are eliminated through the **anus** (CirobuA:0000362), which opens into the atrial chamber. In the branchial chamber, the endostyle (*es* in Fig.5A^I-II^, B-B^I^, D, D^III^) is now characterized by its zones in the form of eight symmetrical longitudinal cellular bands (from the median **zone 1** (CirobuA:0000782) to peripheral **zone 8** (CirobuA:0000790))^1,22^. It is involved in mucus production for filtration. The oral mechanoreceptor, the **coronal organ** (CirobuA:0000923), is developing on the oral tentacles and the velum. It controls the circulating seawater inside the animal body, together with the atrial **cupular organ** (CirobuA:0000924). The **circular muscular system** (CirobuA:0000859) and **longitudinal muscular system** (CirobuA:0000860), responsible for body contraction, become recognizable in the body wall. The heart (*ht* in Fig. 5A^I^, B-B^I^, D^III^), with its inner contractile **myocardium** (*mc* in Fig. 5D^III^; CirobuA:0000886) and outer **pericardium** (*pc* in Fig. 5D^III^; CirobuA:0000679) joined by a **rafe** (*rph* in Fig. 5D^III^; CirobuA:0000887), is now beating.

**Stage 37**

This is the **Early Juvenile I Stage** (CirobuD:0000060; Supplementary Figure S15), which occurs when the endostyle axis is almost parallel to the axis passing through the stalk (63-72 h (3 dpf); Fig. 1B, Stage 37). The stomach swells and the larval tail remnants are no longer present 4 dpf.

The hermaphrodite **reproductive system** (CirobuA:0000909) is now recognizable. The **female reproductive system** (CirobuA:0000910) is formed by a sac-like **ovary** (CirobuA:0000672) continuous in an **oviduct** (CirobuA:0000801). The **male reproductive system** (CirobuA:0000910) comprises the lobular **testis** encrusting the ovary (CirobuA:0000742) and the **sperm duct** (CirobuA:0000920). **Germ cells** (CirobuA:0000916) are maturing within the gonads.

The stalk base forms **test villi** (CirobuA:0000927), each one furnished with a **test vessel** (CirobuA:0000928) in continuity with the haemocele. They ensure firm adhesion to the substrate. In the body, several **blood sinuses** (CirobuA:0000856) can be recognized among organs.

After Stage 37, other territories become histologically recognizable (data not shown). These are the **cloacal cavity** (CirobuA:0000852), the **dorsal languets** (CirobuA:0000636) on the roof of the branchial chamber (CirobuA:0000863), the **pharyngo-epicardial openings** (CirobuA:0000868) putting the **epicardiac cavities** (CirobuA:0000879) in communication with the branchial one, the **endostylar appendix** (CirobuA:0000866), the **oral pigment spots** (CirobuA:0000899) and the **atrial pigment spots** (CirobuA:0000854) encircling the oral and the cloacal siphon border, respectively.

1. Hirano, T. & Nishida, H. Developmental fates of larval tissues after metamorphosis in the ascidian, Halocynthia roretzi. II. Origin of endodermal tissues of the juvenile. *Dev. Genes Evol.* **210**, 55–63 (2000).

2. Nakazawa, K. *et al.* Formation of the digestive tract in Ciona intestinalis includes two distinct morphogenic processes between its anterior and posterior parts. *Dev. Dyn.* **242**, 1172–83 (2013).

3. Imai, J. H. & Meinertzhagen, I. A. Neurons of the Ascidian Larval Nervous System in Ciona intestinalis: I. Central Nervous System. *J. Comp. Neurol.* **501**, 316–334 (2007).

4. Ryan, K., Lu, Z. & Meinertzhagen, I. A. The CNS connectome of a tadpole larva of Ciona intestinalis (L.) highlights sidedness in the brain of a chordate sibling. *Elife* **5**, 1–34 (2016).

5. Moret, F. *et al.* The dopamine-synthesizing cells in the swimming larva of the tunicate Ciona intestinalis are located only in the hypothalamus-related domain of the sensory vesicle. *Eur. J. Neurosci.* **21**, 3043–3055 (2005).

6. Razy-Krajka, F. *et al.* Monoaminergic modulation of photoreception in ascidian: evidence for a proto-hypothalamo-retinal territory. *BMC Biol* **10**, 45 (2012).

7. Horie, T. *et al.* Regulatory cocktail for dopaminergic neurons in a protovertebrate identified by whole-embryo single-cell transcriptomics. *Genes Dev.* **32**, 1297–1302 (2018).

8. Parrinello, D., Parisi, M., Parrinello, N. & Cammarata, M. Ciona robusta hemocyte populational dynamics and PO-dependent cytotoxic activity. *Dev. Comp. Immunol.* **103**, 103519 (2020).

9. Bostwick, M. *et al.* Antagonistic Inhibitory Circuits Integrate Visual and Gravitactic Behaviors. *Curr. Biol.* 1–10 (2020). doi:10.1016/j.cub.2019.12.017

10. Matsunobu, S. & Sasakura, Y. Time course for tail regression during metamorphosis of the ascidian Ciona intestinalis. *Dev. Biol.* **405**, 71–81 (2015).

11. Cloney, R. A. Cytoplasmic filaments and morphogenesis: effects of cytochalasin B on contractile epidermal cells. *Zellforsch* **132**, 167–192 (1972).

12. Numakunai, T. Hentai. in *Gendai Doubutsugaku No Kadai vol.5* (ed. Japan, T. Z. S. of) 135–175 (Gakkai Shuppan Center, 1977).

13. Karaiskou, A., Swalla, B. J., Sasakura, Y. & Chambon, J.-P. P. Metamorphosis in solitary ascidians. *Genesis* **53**, 34–47 (2015).

14. Lash, J. W., Cloney, R. A. & Minor, R. R. The effect of cytochalasin B upon tail resorption and metamorphosis in ten species of ascidians. *Biol. Bull.* **145**, 360–72 (1973).

15. Terakubo, H. Q. *et al.* Network structure of projections extending from peripheral neurons in the tunic of ascidian larva. *Dev. Dyn.* **239**, 2278–87 (2010).

16. Hirano, T. & Nishida, H. Developmental Fates of Larval Tissues after Metamorphosis in AscidianHalocynthia roretzi. *Dev. Biol.* **192**, 199–210 (1997).

17. Chiba, S., Sasaki, A., Nakayama, A., Takamura, K. & Satoh, N. Development of Ciona intestinalis juveniles (through 2nd ascidian stage). *Zoolog. Sci.* **21**, 285–298 (2004).

18. Davidson, B. Ciona intestinalis as a model for cardiac development. *Semin. Cell Dev. Biol.* **18**, 16–26 (2007).

19. Davidson, B. & Levine, M. Evolutionary origins of the vertebrate heart: Specification of the cardiac lineage in Ciona intestinalis. *Proc. Natl. Acad. Sci. U. S. A.* **100**, 11469–73 (2003).

20. Stolfi, A. *et al.* Early Chordate Origins of the Vertebrate Second Heart Field. *Science (80-. ).* **329**, 565 (2010).

21. Wang, W., Razy-Krajka, F., Siu, E., Ketcham, A. & Christiaen, L. NK4 Antagonizes Tbx1/10 to Promote Cardiac versus Pharyngeal Muscle Fate in the Ascidian Second Heart Field. *PLoS Biol.* **11**, e1001725 (2013).

22. Burighel, P., Cloney, R. A. & Cloney, B. Microscopic Anatomy of Invertebrates, Vol. 15. *Microsc. Anat. Invertebr.* **15**, 221–347 (1997).

23. Kawamura, K. *et al.* Germline cell formation and gonad regeneration in solitary and colonial ascidians. *Dev. Dyn.* **240**, 299–308 (2011).

24. Shirae-Kurabayashi, M. *et al.* Dynamic redistribution of vasa homolog and exclusion of somatic cell determinants during germ cell specification in Ciona intestinalis. *Development* **133**, 2683–93 (2006).

25. Stolfi, A. *et al.* Divergent mechanisms regulate conserved cardiopharyngeal development and gene expression in distantly related ascidians. *Elife* **3**, e03728 (2014).

26. Hozumi, A., Horie, T. & Sasakura, Y. Neuronal map reveals the highly regionalized pattern of the juvenile central nervous system of the ascidian Ciona intestinalis. *Dev. Dyn.* **244**, 1375–1393 (2015).

27. Mackie, G. O., Burighel, P., Caicci, F. & Manni, L. Innervation of ascidian siphons and their responses to stimulation. *Can. J. Zool.* **84**, 1146–1162 (2006).

28. Manni, L., Agnoletto, A., Zaniolo, G. & Burighel, P. Stomodeal and neurohypophysial placodes in Ciona Intestinalis: insights into the origin of the pituitary gland. *J. Exp. Zool. Part B Mol. Dev. Evol.* **304B**, 324–339 (2005).

29. Veeman, M. T., Newman-Smith, E., El-Nachef, D. & Smith, W. C. The ascidian mouth opening is derived from the anterior neuropore: reassessing the mouth/neural tube relationship in chordate evolution. *Dev. Biol.* **344**, 138–49 (2010).

30. Ogasawara, M. & Satoh, N. Isolation and Characterization of Endostyle-Specific Genes in the Ascidian Ciona intestinalis. *Biol. Bull.* **195**, 60–69 (1998).

**Supplementary Table S3**

File name: Supplementary File 2

File format: .xlsx

Title of data: Table of developmental stages in *Ciona*

Description of data: Table listing the Meta-Periods, Periods, and Stages of development of *Ciona*, from Stage 0 (unfertilized egg) to Stage 43 (adult). Main features, time of appearance after fertilization, and comparison with the staging method by Chiba and collaborators (Chiba et al., 2004) are reported for each stage. Yellow: stages defined in this work; Stages 26-37 are described in this work.

**Supplementary Video S4**

File name: Supplementary File 3

File format: .mov

Title of data: *C. robusta* (*C. intestinalis* type A) development

Description of data: Time-lapse movie showing the development of *Ciona* from the fertilized egg (Stage 1) to the juvenile (Stage 38). Observation was performed at 18°C for Stages 1-26 and at 20°C for Stages 27-37.

**Supplementary Figure S5**

Title of data: Three-dimensional reconstructed images of *C. robusta* (*C. intestinalis* type A) post hatching stages

Description of data: Specimens labeled with Alexa 546 phalloidine (Molecular Probes). As the staining targets actin filaments, the cortical cytoplasm is stained in each cell. Stages 26-33: left view, anterio at left. Stages 34-37: leftview, anterior (oral siphon) at right (Stages 34-35) or top (Stages 36-37). Scale bar: 50 μm. CLSM.


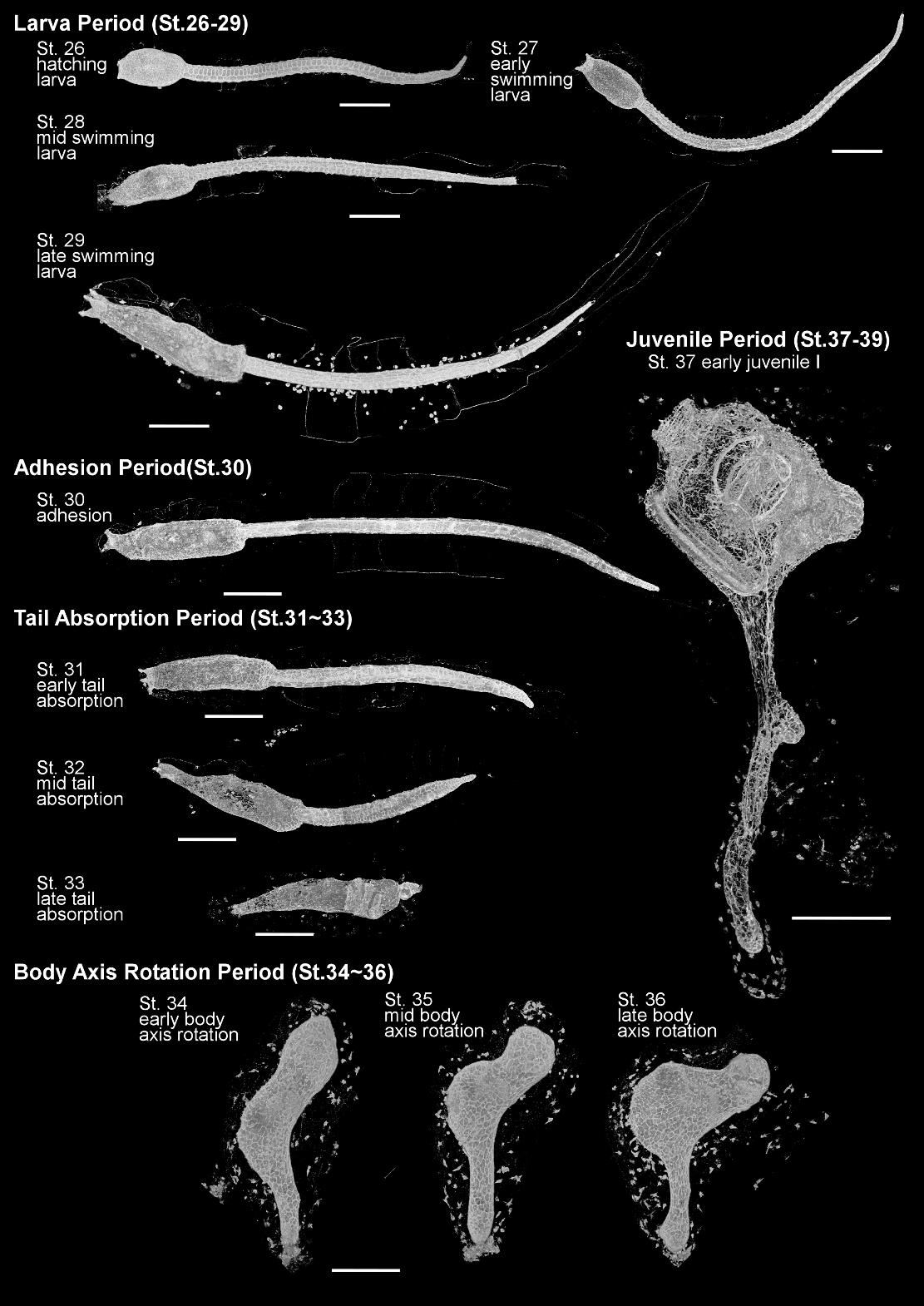


**Supplementary Data S6**

File name: Supplementary File 4

File format: .xls

Title of data: List of references included in the ontology

Description of data: References used to build up and annotate in the AO and the DO of *C. robusta* (*C. intestinalis* type A).

**Supplementary Data S7**

File name: Supplementary File 5

File format: .xls

Title of data: Anatomical Ontology of *C. robusta* (*C. intestinalis* type A) from Stage 26 to Stage 37

Description of data: Table listing the anatomical entities of the AO (Columns B-F: Terms and their specifications), their definition (Column G), Part of (Column H), Develops from (Column I), End Stage (Column J), Start Stage (Column K), Comment (Column L), Literature (Column M), and ID (Column N).

**Supplementary Data S8**

File name: Supplementary File 6

File format: .xls

Title of data: List of anatomical entities, abbreviations, and definitions

Description of data: Table listing, in alphabetical order, the anatomical entities of the AO, their abbreviations used in Figures and Supplementary files, and their definitions.

**Supplementary Figure S9**

Title of data: Hatching larva (Stage 26). CLSM

Description of data: **A.** Larva in the left lateral view. DCEN, RTEN, VCEN: cilia to dorsal caudal, rostral trunk, and ventral caudal epidermal neurons, respectively; pp: three anterior adhesive papillae. **B-B^I^.** Medial sagittal optic sections of the larval trunk (B) and posterior part of the tail (B^I^). Enlargement is the same in B and B^I^. **C-C^I^.** Transverse optic sections of larval trunk at sensory vesicle (C) and the atrial siphon primordia (C^I^) level. Enlargement is the same in C and C^I^. **D-D^I^.** Frontal optic sections of the larval trunk at the sensory vesicle (D) and ventral pharynx (D^I^) level. Enlargement is the same as C. ant pha: anterior pharynx; CNS: larval central nervous system; epi: epidermis; lasp: left atrial siphon primordium; mech: mesenchyme; ne: neck; nc: nerve cord; noto: notochord; oc: ocellus; osp: oral siphon primordium; noto: notochord; pha: pharynx; post pha: posterior pharynx; pp: papilla; rasp: right atrial siphon primordium; sv: sensory vesicle; vg: visceral ganglion.


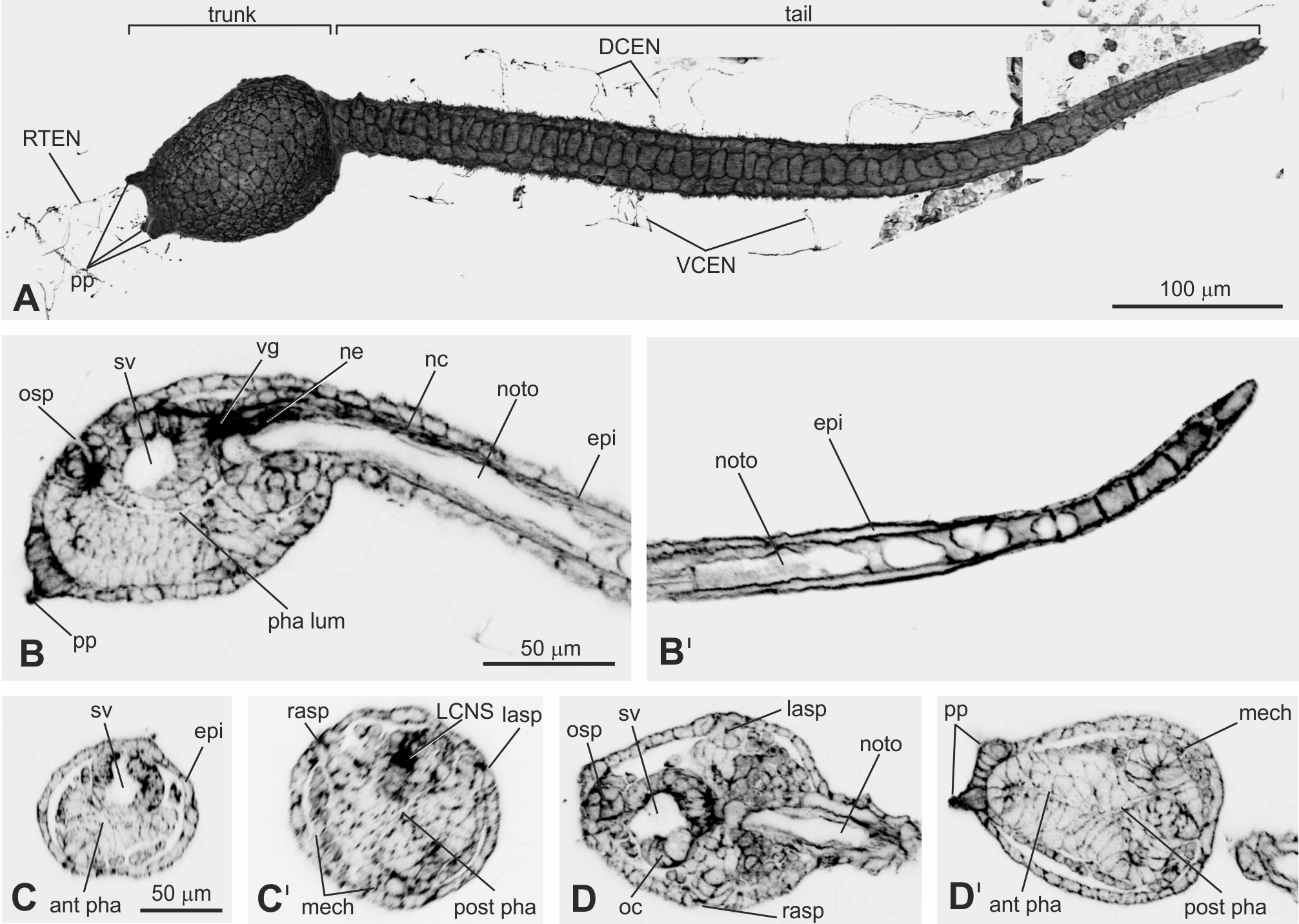


**Supplementary Figure S10**

Title of data: Mid swimming larva (Stage 28)

Description of data: **A.** Larva, left view. Note the ciliary network belonging to the epidermal sensory neurons (DCEN, RTEN, VCEN: cilium of a dorsal caudal, rostral trunk, and ventral caudal epidermal neuron, respectively). CLSM. **B.** Median sagittal optic section. CLSM. **C-C^II^.** Two transverse optic sections of the same larva of B. Enlargement is the same in D-D^I^. CNS: central nervous system; epi: epidermis; lasp: left oral siphon primordium; mech: mesenchyme; nc: nerve cord; nd: neurohypophyseal duct; ne: neck; noto: notochord; pl: preoral lobe;; osp: oral siphon primordium; pha: pharynx; pp: dorsal papillae; sv: sensory vesicle; vg: visceral ganglion.


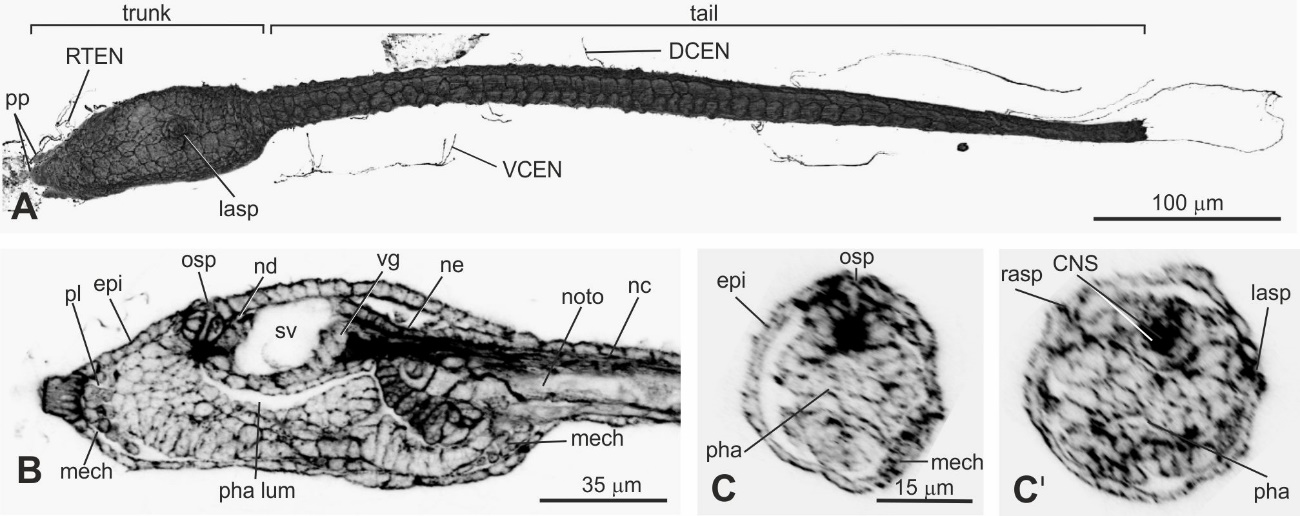


**Supplementary Figure S11**

Description of data: **A.** Larva, left view. CLSM. ATEN, DCEN, RTEN, VCEN: cilium of an anterior trunk, dorsal caudal, rostral trunk, and ventral caudal epidermal neuron, respectively. **B.** Medial sagittal optic section of the larval trunk. CLSM. **C-C^I^.** Two frontal sections of the same larva, at the level of atrial siphon primordia (C) and endodermal strand (esr) (C^I^). Light microscopy, Toluidine blue. Enlargement is the same in **C-C^I^**. **D-D^VII^.** Eight selected transverse sections from a complete dataset of a serially sectioned larva from anterior (D) to posterior (D^VII^). Light microscopy, Toluidine blue. Enlargement is the same in D-D^VII^. CNS: central nervous system; cor: coronet cells; epi: epidermis; esp: endostyle primordium; iclt (C2) and oclt (C1): inner (C2) and outer (C1) cuticular layer of the tunic, respectively; gp: gut primordium; lasp: left atrial siphon primordium; mech: mesenchyme; nc: nerve cord; noto: notochord; oc: ocellus; ot: otolith; pha lum: pharynx lumen; pl: preoral lobe; pp: ventral papilla; rasp: right atrial siphon primordium; sv: sensory vesicle; tc: test cell; tf: tail fin; tmc: tail muscle cells; tun: tunic; tunc: tunic cells; vg: visceral ganglion.


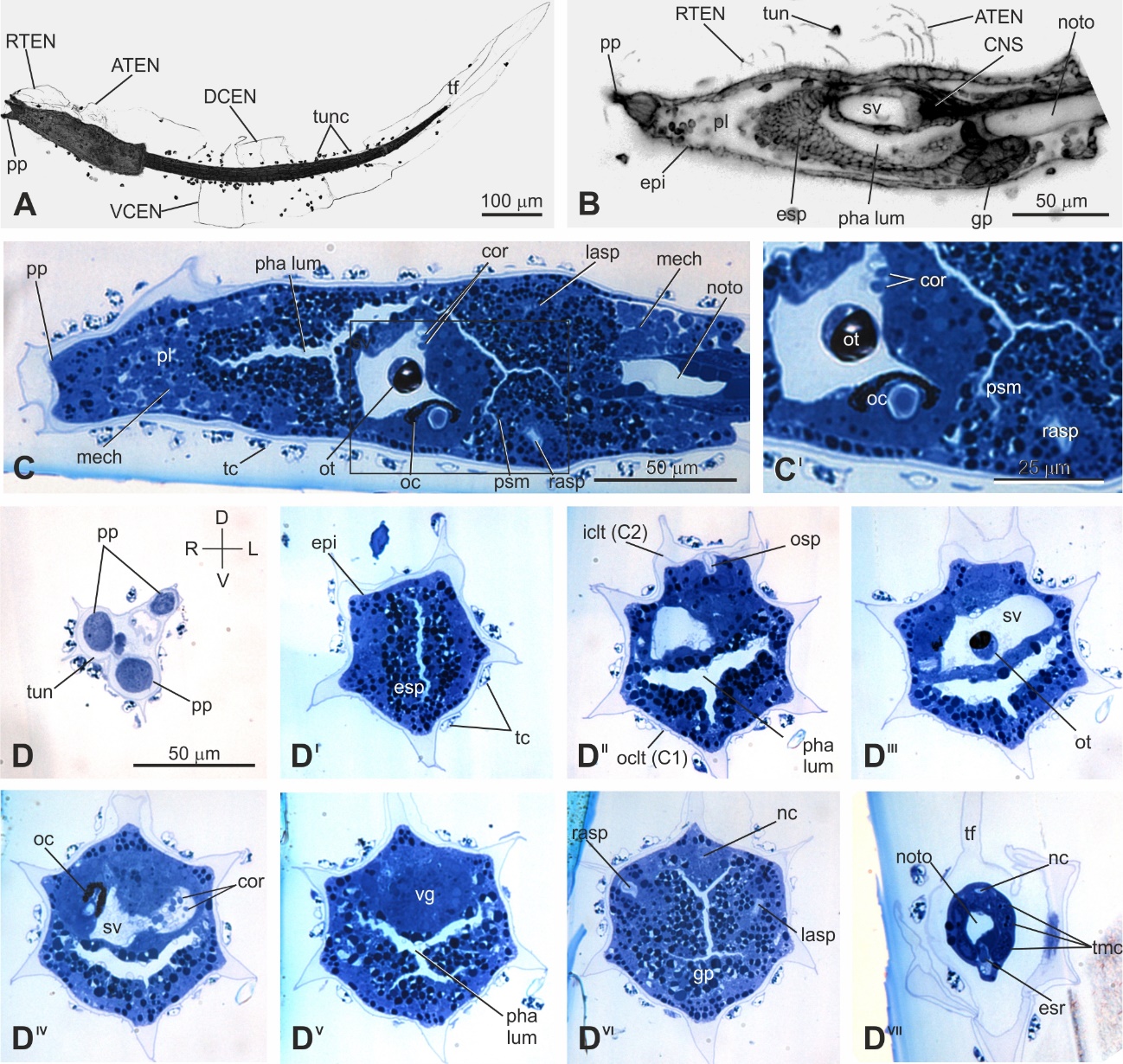


**Supplementary Figure S12**

Title of data: Early tail absorption (Stage 31) (A-B^I^) and mid tail absorption (Stage 32) (C-F^II^). CLSM

Description of data: **A.** Larva in early tail absorption, seen from the left side. **B-B^I^.** Medial sagittal optic sections of the trunk (B) and tail tip (B^I^) of the larva shown in A. **C.** Larva in the mid-tail absorption, seen from the left side. The line on the larval trunk labeled by D^II^ indicates the level of the transverse section shown in D^II^. **D-D^I^.** Medial sagittal optic sections of the tail tip (D) and trunk (D^I^) of the larva shown in C. In D^I^, the line on the larval trunk labeled by D^II^ indicates the level of transverse section shown in D^II^. Enlargement is the same in D-D^I^. **D^II^.** Transverse optic section of the larval trunk shown in C and D^I^. **E-E^II^**. Larva in mid-tail absorption (E), seen from the left side, at a slightly more advanced stage than in C, and detail of its tail tip (E^I^, and its optic section in E^II^). The lines on the larval trunk labeled by F and F^II^ indicate the levels of sections shown in F and F^II^, respectively. Enlargement is the same in E-E^II^. **F-F^II^.** Transverse (F), medial sagittal (F^I^), and frontal (F^II^) optic sections of the trunk of larva shown in E. Enlargement is the same in F ^I^-F^II^. abs tail: absorbing tail; ATEN, DCEN, RTEN, VCEN: cilium of an anterior trunk, dorsal caudal, rostral trunk, and ventral caudal epidermal neuron, respectively; deg pp: degenerating papilla; deg tail: degenerating tail; esr: endodermal strand; epi: epidermis; esp: endostyle primordium; gp: gut primordium; ht: heart; lasp: left atrial siphon primordium; lbr: larval brain remnants; mech: mesenchyme; oes: oesophagus; ne: neck; noto: notochord; pha lum: pharynx lumen; pl: preoral lobe; pp: ventral papilla; rasp: right atrial siphon primordium; sv: sensory vesicle; tf: tail fin; tmc: tail muscle cell; tunc: tunic cells; vg: visceral ganglion.


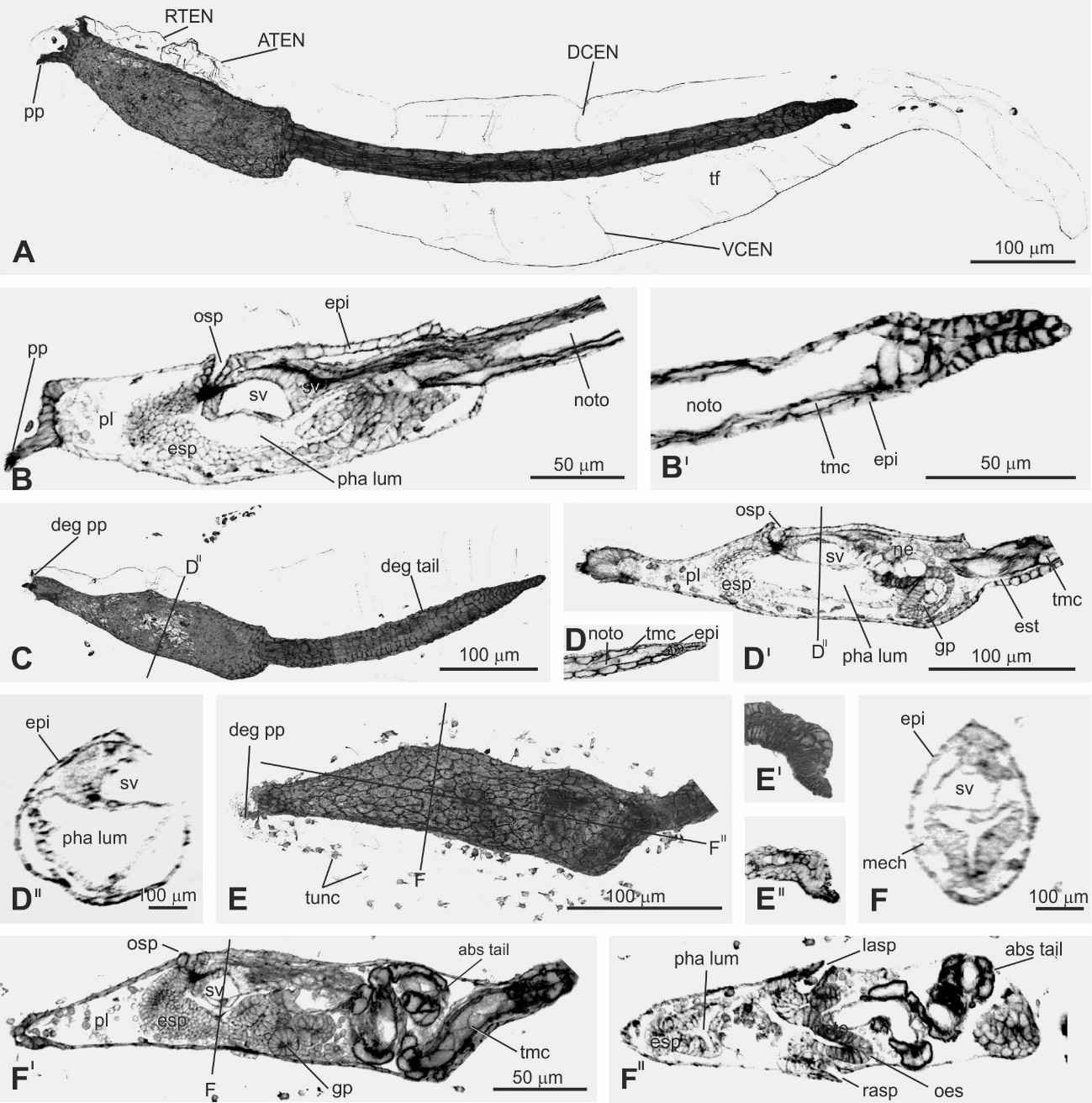


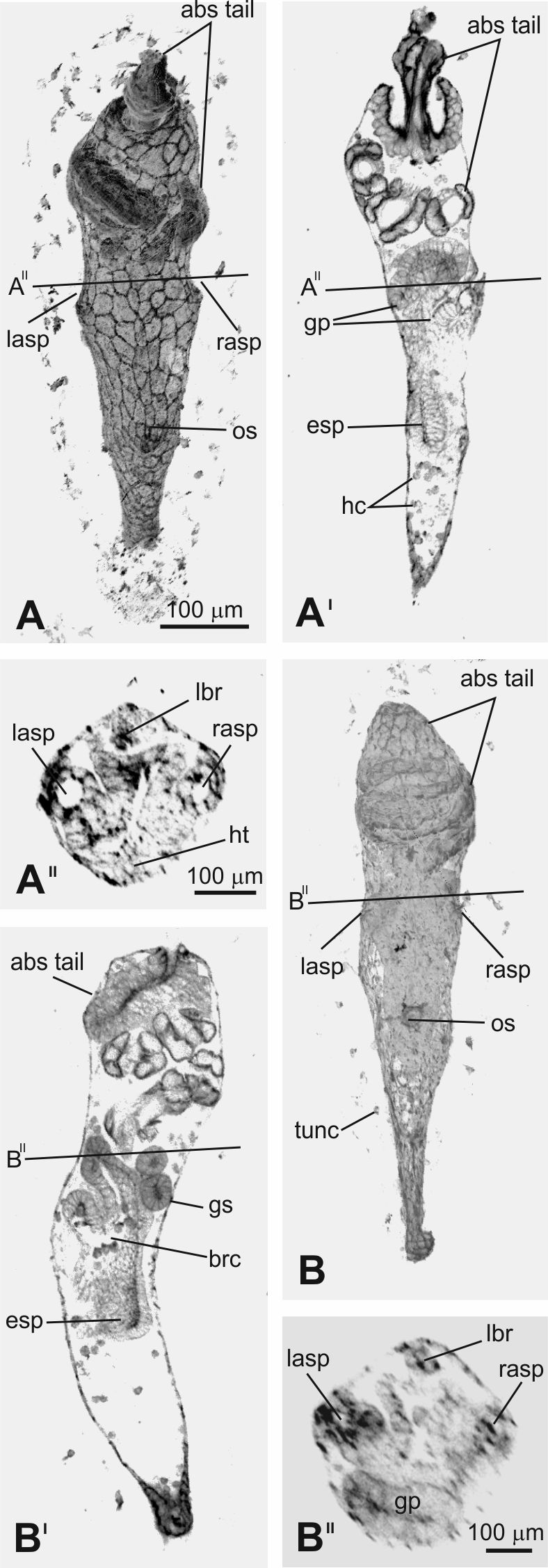
**Supplementary Figure S13**

Title of data: Late tail absorption (Stage 33). CLSM

Description of data: **A-A^II^.** Larva in late tail adsorption (A), seen from the dorsal side, and optic frontal (A^I^) and transverse (A^II^) sections. The line on the larval trunk labeled by A^II^ indicates the level of the section shown in A^II^. Enlargement is the same in A-A^I^. **B-B^II^.** Larva in late tail absorption (B), seen from the dorsal side at a more advanced stage than in A, and optic frontal (B^I^) and transverse (B^II^) sections. The line on the larval trunk labeled by B^II^ indicates the level of the section shown in B^II^. Enlargement in B-B^I^ is the same as in A. abs tail: absorbing tail; brc: branchial chamber; esp: endostyle primordium; gp: gut primordium; gs: gill slit; hc: haemocyte; lasp: left atrial siphon primordium; os: oral siphon; rasp: right atrial siphon primordium.

**Supplementary Figure S14**

Title of data: Mid body axis rotation (Stage 35)

Description of data: **A-A^IV^.** Metamorphosing larva, seen from the left side (A) and its medial sagittal (A^I^), frontal (A^II^), and transverse (A^III^) optic sections. A^IV^ is an enlargement of the squared area in A^I^. In A^I^, the lines on the larval trunk labeled by A^II^ and A^III^ indicate the levels of sections shown in A^II^ and A^III^, respectively. brc: branchial chamber; cil duc: ciliated duct of neural gland; es: endostyle; hc: haemocytes; ht: heart; las: left atrial siphon; lpsm: left protostigmata; ng: neural gland; os: oral siphon; osm: oral siphon muscle; psm: right protostigmata; stom: stomach; tail remn: tail remnants; tun: runic; tunc: tunic cells. Enlargement is the same in A and A^I^.


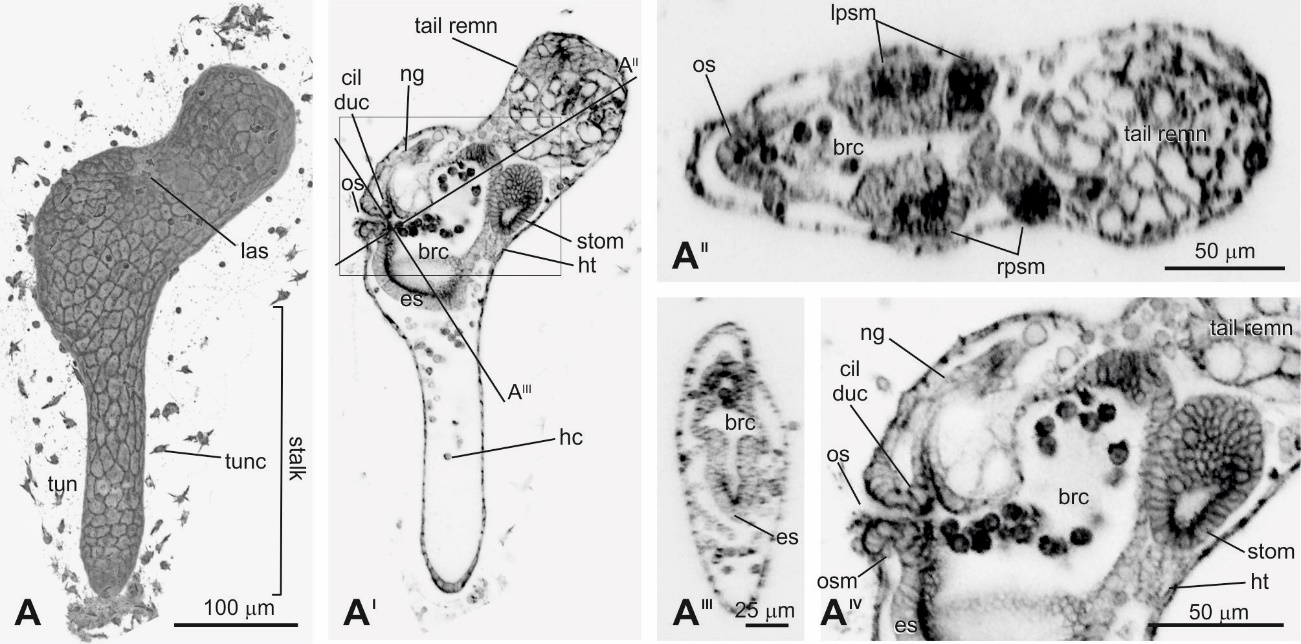


**Supplementary Figure S15**

Title of data: Early juvenile I (Stage 37)

Description of data: **A-A^II^.** Juvenile, seen from the left side (A), its medial sagittal optic section (A^I^) and its depth-coded image. The depth information is represented by a heat map: warmer colors go to the front, and cooler colors to the back. For the color bar, see Fig. 5A^II^. Lines indicated by B-B^VI^ in A represent the level of transverse sections shown in B-B^VI^.CLSM. Enlargement is the same in A-A^II^. **B-B^VI^.** Transverse sections of a juvenile from the dorsal (B) to ventral (B^VI^) sides. Squared areas in B^III^ and B^IV^ are enlarged in insets to show details of endostyle (B^III^), left atrial siphon (black square), and row of ciliated cells of a protostigma (red square) (B^IV^), respectively. Numbers 1-8 in the inset of B^III^ indicate the eight zones of endostyle. Arrowheads in B^II^: ciliated cells of the coronal organ; asterisks in B^III^-B^IV^: protostigmata. Toluidine blue. Enlargement is the same in B-B^VI^. as: atrial siphon; brc: branchial chamber; cil duc: ciliated duct of neural gland; cg: cerebral ganglion; cut: tunic cuticle; dl: dorsal lamina; es: endostyle; hc: haemocyte; int: intestine; las: left atrial siphon; lasm: left atrial siphon muscles; lbr: larval brain remnant; man: mantle; mc: myocardium; mint: medium intestine; ng: neural gland; oes: oesophagus; os: oral siphon; osm: oral siphon muscle; pb: peripharyngeal band; pc: pericardium; pg: pyloric gland; prox int: proximal intestine; psm: protostigmata; pyc: pyloric caecum; stom: stomach; term int: terminal intestine, close to anus; ras: right atrial siphon; tail remn: tail remnants; ten: oral tentacles; tun: runic; tunc: tunic cells. **
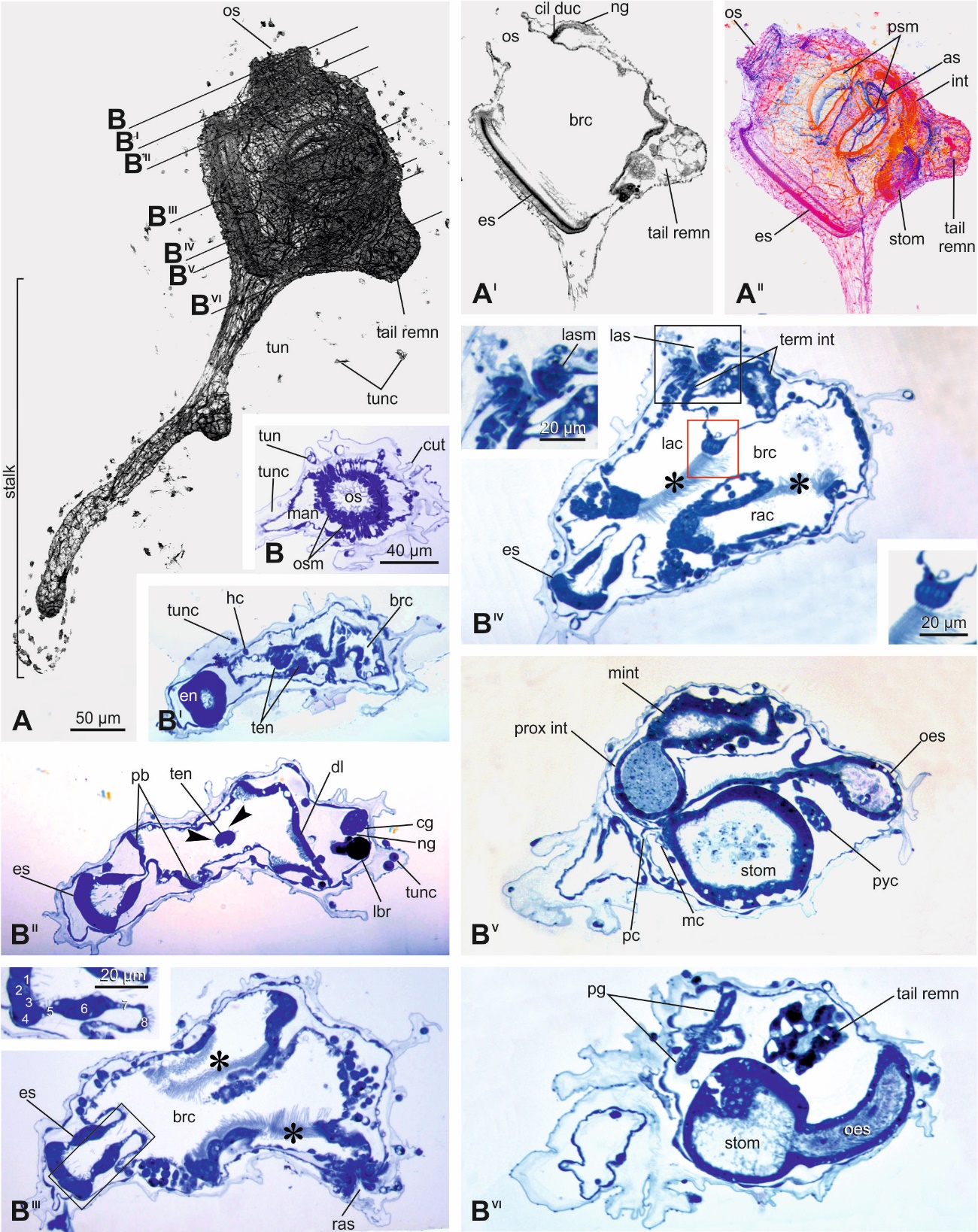
**

**Supplementary Table S16**

Title of data: List of anatomical ontologies

Description of data: Table of available ontologies regarding the anatomy of animal model organisms summarizing: the combination of AO with developmental stages, the number of anatomical terms listed, the percentage number of the relation “develops from” with respect to the total number of terms, the use of references as a source of data, and the link to the FAIRsharing database.


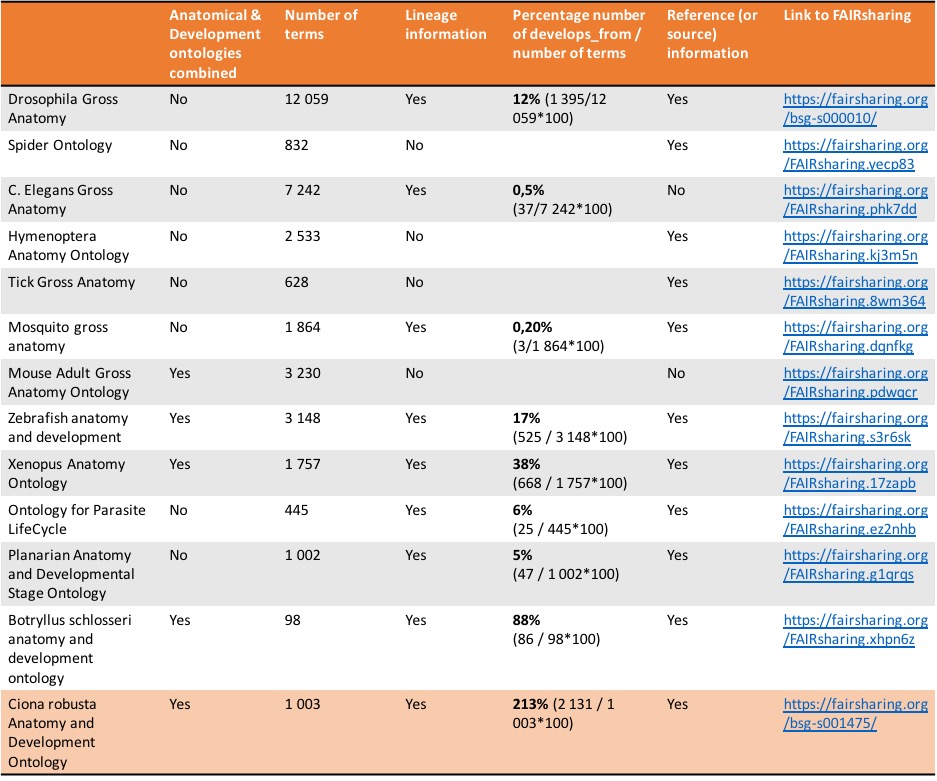

Supplement: Supplementary file 1 — Supplementary information 1 [file 41598_2020_73544_MOESM1_ESM.docx]
